# Physical basis of the ‘magnification rule’ for standardized Immunohistochemical scoring of HER2 in breast and gastric cancer

**DOI:** 10.1186/s13000-018-0696-x

**Published:** 2018-03-12

**Authors:** Andreas H. Scheel, Frédérique Penault-Llorca, Wedad Hanna, Gustavo Baretton, Peter Middel, Judith Burchhardt, Manfred Hofmann, Bharat Jasani, Josef Rüschoff

**Affiliations:** 1 Institute of Pathology, University Hospital Cologne, Kerpener Str. 62, 50937 Cologne, Germany; 2 Département de Pathologie, Centre Jean-Perrin, 58, rue Montalembert, 392, 63011 Clermont-Ferrand cedex 1, BP France; 3 Department of Laboratory Medicine and Pathobiology, University of Toronto, Toronto, Canada; 4 Institute of Pathology, University Hospital Dresden, Fetscherstr, 74, 01307 Dresden, Germany; 5 Institute of Pathology Nordhessen, Germaniastraße 7, 34119 Kassel, Germany; 6 Institute of Pathology, University Hospital Göttingen, Robert-Koch-Str. 40, 37075 Göttingen, Germany; 7 Targos Molecular Pathology GmbH, Germaniastraße 7, 34119 Kassel, Germany

**Keywords:** HER2/neu, Immunohistochemistry, Breast cancer, Gastric cancer, Magnification rule, Predictive biomarker

## Abstract

**Background:**

Detection of HER2/neu receptor overexpression and/or amplification is a prerequisite for efficient anti-HER2 treatment of breast and gastric carcinomas. Immunohistochemistry (IHC) of the HER2 protein is the most common screening test, thus precise and reproducible IHC-scoring is of utmost importance. Interobserver variance still is a problem; in particular in gastric carcinomas the reliable differentiation of IHC scores 2+ and 1+ is challenging.

Herein we describe the physical basis of what we called the ‘magnification rule’: Different microscope objectives are employed to reproducibly subdivide the continuous spectrum of IHC staining intensities into distinct categories (1+, 2+, 3+).

**Methods:**

HER2-IHC was performed on 120 breast cancer biopsy specimens (*n* = 40 per category). Width and color-intensity of membranous DAB chromogen precipitates were measured by whole-slide scanning and digital morphometry. Image-analysis data were related to semi-quantitative manual scoring according to the magnification rule and to the optical properties of the employed microscope objectives.

**Results:**

The semi-quantitative manual HER2-IHC scores are correlated to color-intensity measured by image-analysis and to the width of DAB-precipitates. The mean widths ±standard deviations of precipitates were: IHC-score 1+, 0.64 ± 0.1 μm; score 2+, 1.0 ± 0.23 μm; score 3+, 2.14 ± 0.4 μm. The width of precipitates per category matched the optical resolution of the employed microscope objective lenses: Approximately 0.4 μm (40×), 1.0 μm (10×) and 2.0 μm (5×).

**Conclusions:**

Perceived intensity, width of the DAB chromogen precipitate, and absolute color-intensity determined by image-analysis are linked. These interrelations form the physical basis of the ‘magnification rule’: 2+ precipitates are too narrow to be observed with 5× microscope objectives, 1+ precipitates are too narrow for 10× objectives. Thus, the rule uses the optical resolution windows of standard diagnostic microscope objectives to derive the width of the DAB-precipitates. The width is in turn correlated with color-intensity. Hereby, the more or less subjective estimation of IHC scores based only on the staining-intensity is replaced by a quasi-morphometric measurement. The principle seems universally applicable to immunohistochemical stainings of membrane-bound biomarkers that require an intensity-dependent scoring.

**Electronic supplementary material:**

The online version of this article (10.1186/s13000-018-0696-x) contains supplementary material, which is available to authorized users.

## Background

Targeting the HER2/neu pathway [1] has shown remarkable efficiency in the treatment of breast and gastric cancer [2, 3]. A prerequisite for specific treatment is the demonstration of HER2 receptor overexpression by immunohistochemistry (IHC) and/or *HER2/neu* gene amplification by in-situ hybridization (ISH) [4–6]. Although advanced DNA-sequencing techniques have been demonstrated to analyze panels of oncogenic genomic aberrations including amplification of HER2/neu [7], current testing guidelines are based on IHC and ISH only [4, 5]. Most algorithms use IHC as first screening test and ISH as second test for the confirmation of equivocal cases (IHC 2+). Thus, IHC plays a key-role for HER2 testing in the routine diagnostics of breast and gastroesophageal cancer.

Interpretation of HER2-IHC is, however, more or less subjective which causes overall disagreement rates of around 10% [8]. The main issue in breast cancer is false positive scoring while in gastric cancer false negative scoring is the major problem. In a retrospective central review of 187 HER2 stained breast cancer specimens from 10 pathological institutions 9.5% of the negative cases were reclassified as positive and 31.7% of the positive cases as negative [9]. In gastric cancer, a central review of 394 HER2 stained specimens from 19 French pathological institutions revealed a false positive rate of 5% but a false negative rate of 27.4% [10]. This problem has recently also been addressed by the panelists of the new HER2 testing guideline for gastric and gastroesophageal cancer [5]. It is stated that in particular reproducibility of 1+ and 2+ IHC scores can be low and the distinction between 1+ and 2+ is “challenging”. However, it remains unclear to the reader how this particular scoring problem can be resolved in clinical practice.

From the perspective of our long-standing experience with HER2 testing, e.g., as the central lab for HERA [2] and ToGA [3] trials, we consider subjectivity in IHC-scoring as major source of discordant results between local and central testing. This is particularly true for false negative HER2 testing in gastric cancer. In contrast to breast cancer where ring-shaped membranous staining is crucial to score a case either positive (IHC 3+) or potentially positive (IHC2+), scoring in gastric cancer is solely based on intensity assessment by eye. Due to neurophysiological limitations it is practically impossible to objectively assess color-intensities alone unless other structural criteria, e.g. such as ring-shaped staining, are included [11–13].

In the context of the ToGA-study [3] we therefore developed a semiquantitative approach called ‘magnification rule’ (MR) that relates staining-intensity to the microscope magnification used to perceive it: Any membranous staining that can be recognized at low magnification (2.5-5× objective lens) corresponds to IHC3+; if higher magnification (10×-20×) is needed to unequivocally identify stained membranes, IHC2+ is diagnosed. Any staining visible only at 40× objective lens represents an IHC1+ score [14, 15].

By using this rule the inter-observer consensus raised significantly from κ< 0.5 to κ=0.805 in a study on 547 gastric cancer specimens evaluated by six pathologists [15]. The finding was confirmed by a recent study which compared HER2 scoring by conventional light microscopy and by virtual microscopy and yielded inter-observer concordance values of up to κ=0.811 [16]. Thus, the MR has already been incorporated in national recommendations on HER2-testing in gastric cancer [6, 17]. This quasi-morphometric semiquantitative approach applies also to HER2-IHC scoring in breast cancer where it is used for the first step of scoring, i.e. the estimation of the color-intensity, before the second criterion, the ring-shape pattern of the staining, is assessed [15, 17].

The present study analyses the physical background of the MR using a series of 120 breast cancer samples immunostained for HER2. The data provide a physical basis of how the MR works to overcome subjectivity in the scoring of membrane-bound IHC-biomarkers.

## Methods

### Breast cancer biopsy specimens

**Fig. 1:**
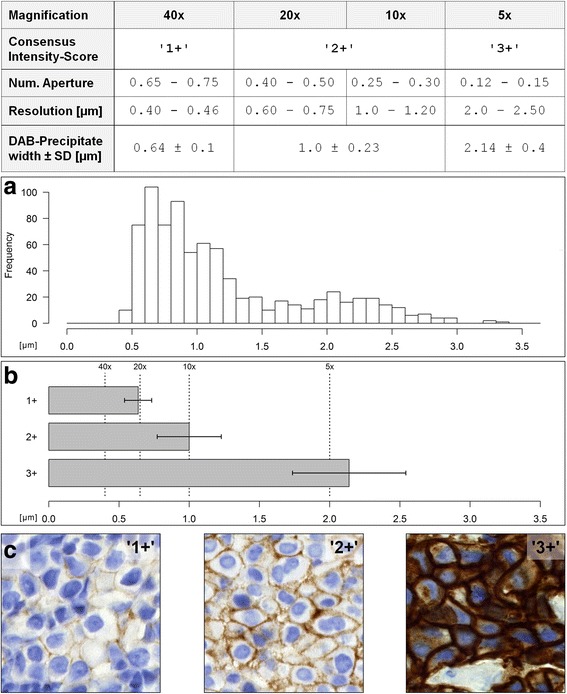
Her2-IHC scoring categories reflect DAB-precipitate widths. Table: Microscope objectives have a fixed resolution that depends on the numerical aperture (Range: Values of common objectives). DAB-precipitates in HER2-IHC differ in width according to the intensity score. **a** histogram: Summary of 1200 DAB-precipitate-width measurements in μm. **b** bar chart: Mean DAB-width (bars) ±SD (antennae); resolution of standard microscope objectives (dashed lines). **c** images: Representative HER2-IHC stainings of invasive ductal breast carcinomas according to intensity score

One hundred and twenty specimens of invasive breast carcinoma (no special subtype; NST) were retrospectively investigated using routinely HER2 stained biopsies diagnosed within one year at the Institute of Pathology Nordhessen, Kassel, Germany (Example photomicrographs: Fig. 1, Additional file 1: Figure S1). HER2 status was determined according to the 2013 updated ASCO/CAP recommendations [4]. Accordingly, carcinomas classified as IHC 2+ were subsequently tested with dual-color chromogenic in situ hybridization (ISH) for amplification of the HER2/Neu Gene (INFORM HER2 Dual ISH DNA Probe Cocktail Assay, Ventana Medical Systems Inc., Tucson, USA). Anonymized cases were scored by three pathologists and the consensus score for each taken as the final IHC HER2 status.

### IHC-staining and digital quantification

Immunohistochemistry (IHC) was performed using the 4B5 anti-HER2 primary antibody and a polymer-based detection system (UltraView DAB) on a BenchMark automated staining system (all by Ventana Medical Systems Inc., Tucson, USA). Peroxidase-conjugated secondary antibodies were used for chromogenic detecting by oxidizing 3,3′-Diaminobenzidin according to the manufactures protocol.

IHC HER2 stained slides were digitized using a Pannoramic P250 whole slide scanner (3D Histech, Budapest, Hungary) at 5.11 pixel/μm. DAB-precipitate thickness was measured with ‘ImageJ’ image-analysis software [18]. The regions of interest (ROIs) were manually defined according to the following rules: 10 non-adjacent tumor cells were measured per specimen. For each cell, ROIs perpendicular to the precipitate were drawn at 4 positions, i.e. 40 measurements per specimen, 4800 measurements in total. DAB-precipitate intensities were calculated using a color deconvolution algorithm [19]. The mean ROI-length and color intensity was calculated for each cell. The 8bit grey-scale intensity-values (0 = black, 256 = white) are as stated inverted, relative values to facilitate interpretation (0% = white, no staining; 100% black, full staining-intensity).

### Microscope resolution

The resolution (d) of the objective of a light microscope is the minimum distance required to distinguish two adjacent points on a focal plane. In light microscopy d is determined by the numerical apertures (NA) of the microscope objectives and condenser and the wavelength of the employed light (λ) through Abbe’s law $=\frac{\uplambda}{2 NA}$ . *NA* is defined as *NA* = *n* ∗ sin(*α*) by the half-angle of the maximum cone of light that can pass through the objective (α) and the index of refraction (*n*) of the medium in which the objective is used, $d=\frac{\uplambda}{2n\sin \left(\alpha \right)}$ . For λ=600 nm, standard diagnostic microscope objectives yield the resolutions 5×: 2.0 μm (*NA*=0.14), 10×: 1.0 μm (*NA*=0.3), 20×: 0.6 μm (*NA*=0.5), 100×: 0.4 μm (*NA*=0.75).

### Statistics

Statistics and statistical testing were performed using ‘R’ statistical programming language (http://www.r-project.org/). The data were found normally distributed and were tested using the Welch two-sample t-test. In all tests, the significance level was set to α = 1%.

## Results

### HER2-IHC scoring categories reflect the width of DAB-precipitates

In total, *n* = 120 cases of invasive breast carcinoma (no special subtype; NST) were analyzed which yielded 4800 individual measurements. The linear DAB-precipitates formed by the HER2-IHC were quantified. Plotting the width of the precipitates per cells as continuous histogram yielded a biphasic distribution (Fig. 1a). However, if the cells per case are aggregated by the arithmetic mean, three groups emerged that matched the manual scoring categories (Fig. 1b, Additional file 2: Figure S2). The mean widths were found to be: IHC-score 1+, 0.64 ± 0.1 μm; score 2+, 1.0 ± 0.23 μm; score 3+, 2.14 ± 0.4 μm. The differences between the three groups are statistically significant (*p* < 0.01). Thus, the scoring categories indicate groups of cases with perceivable differences in the widths of the DAB-precipitates.

The values were related to the optical resolutions of diagnostic microscope objectives. As predicted by the MR, precipitates of the scoring category 1+ are too narrow to be observable with a 10× microscopic lens and are delineated best by a 40× objective. Moreover, 2+ precipitates are broad enough to be visible at 10× but to narrow to be visible at 5×. Only 3+ precipitates were found broad enough to be readily recognizable if a 5× (or even a 2.5×) objective lens is used (Fig. 1). The forth scoring category, ‘0’ was omitted, as the DAB-precipitates were found absent or insufficient for quantification.

### Precipitate width and color intensity are correlated

**Fig. 2:**
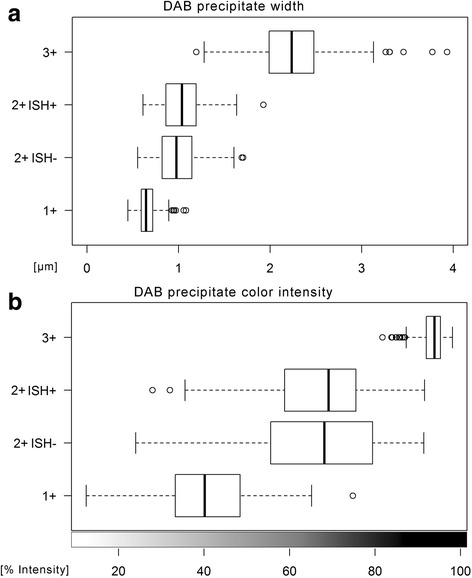
Width and color intensity of HER2 are correlated. Immunohistochemistry of *n* = 120 cases of invasive ductal carcinoma of the mammary gland show that width of the linear DAB precipitates (**a**) and DAB color intensity (**b**) are correlated (8bit scale, inverted relative values). However, neither parameter discriminates IHC Score 2+ samples that are *Her2* amplified (ISH+) or not amplified (ISH-) by in situ hybridization

Color intensity of the DAB-precipitates was determined using color deconvolution [19]. Similar to the precipitate width, the intensities of the three scoring categories were significantly different (*p* < 0.01) (Fig. 2). Moreover, a good linear correlation between width and intensity was noticed among scoring categories 1+ and 2+ (Pearson’s *r* = 0.73). The intensity in scoring category 3+ was saturated (Additional file 3: Figure S3).

### Precipitate width and color intensity do not differentiate between amplified and non-amplified cases in the IHC 2+ scoring category

All cases classified as IHC 2+ were subsequently tested for *HER2/neu* gene amplification by in situ hybridization (ISH). Among the study cases, 20 were ISH positive and 20 were ISH negative. The DAB-precipitate width showed a non-significant difference between the ISH positive cases (1.02 ± 0.23 μm) and the ISH negative cases (0.98 ± 0.22 μm, *p* = 0.02485). Indeed, histograms of the individual cells showed that the IHC 2+ ISH positive and IHC 2+ ISH negative cases feature almost completely overlapping precipitate widths (Additional file 2: Figure S2). No difference in the HER2-IHC color intensities was noticed between ISH negative and ISH positive cases either (*p* = 0.7493) (Fig. 2).

## Discussion

HER2-IHC scores determined according to the ‘magnification rule’ (MR) were compared to image-analysis of width and color intensity of the DAB chromogen precipitates along the tumor cell membranes. The parameters were closely correlated and matched the optical resolutions of the employed microscope objectives. This provides a physical basis of the MR which was originally established as an empirical rule for standardized HER2-IHC scoring in gastric cancer.

HER2-IHC assays are based on peroxidase-coupled secondary antibodies that oxidize 3,3′-diaminobenzidine (DAB) into an insoluble, brownish precipitate at the spot of the bound epitope. As HER2 is confined to the cell-membrane, the reaction yields linear precipitates at the cell-boundaries. Technical aspects of HER2-IHC are robust and can be standardized by validated protocols, on-slide control tissue and external quality assessment [20–22] but interpretation of the resulting staining patterns may be challenging [4]: HER2-IHC scoring relies on subdividing the cases into categories based on staining-intensity (0, 1+, negative; 2+, equivocal, requires ISH-testing; 3+ positive) (Fig. 1, Additional file 1: Figure S1).

The human optical system is optimized to notice relative differences in color-intensity rather than absolute values. Visual stimuli are precortically processed in the retina through lateral inhibition which underlies varies optical illusions first described by Ernst Mach in 1865 as ‘Mach bands’ [12]. A given surface might appear brighter or darker depending on the luminosity of its surroundings [11, 13]. Accordingly, intensity-scores in histopathology are in general prone to subjectivity.

This is of particular importance in HER2-IHC scoring in gastric cancer, which is based solely on staining-intensity. In contrast, HER2-IHC scoring in breast cancer also includes the staining-pattern as the DAB-precipitates have to be ring-shaped to be considered as IHC 3+. In particular, inter-observer reproducibility of categories 1+ and 2+ scores can be low in gastric cancer. In two global clinical trials 6.4% (29/455) of cases with intestinal or mixed histology were found erroneously classified as HER2-IHC negative. In most of these cases (*n* = 21) initial scores were IHC 0 or IHC 1+ whereas central counter-testing revealed either IHC 2+ and ISH positivity (*n* = 17) or even IHC 3+ (*n* = 4). Four additional cases locally IHC 2+ FISH-negative were centrally ISH positive [23].

In a recent French study comprising 393 centrally re-evaluated gastric carcinomas false negative rate reached even 27.4% (20/73 HER2-IHC 2+). False positive rate was 5% (16/320) with an overall discordance rate of 9% [10].

In the present study it could be demonstrated that objectively measured widths and color intensities of the linear membranous precipitates correlate with the semiquantitative intensity score manually assessed by MR. Utilizing this approach circumvents the need to interpret the staining just by color-intensity and constitutes a quasi-morphometric measurement.

Our data suggest that the MR might be applicable to other membrane-bound biomarkers as well. Indeed, inter-observer concordance of IHC-scoring of EGFR could be significantly improved within a round-robin test that included 11 international pathological laboratories [24]. The MR could also be included in the comprehensive ‘Histo-Score’ (H-Score) [25]. The H-Score incorporates all IHC-intensity categories and is frequently used to determine an optimal cut-point in IHC-scoring [26, 27]. The prerequisite to using the MR is that biological relevant scoring-categories have to be reflected by differences in the geometry of the histological stain that match the optical resolution windows of the microscope objectives. A given IHC-protocol could be optimized to match the appropriate intensity range.

The interrelation of DAB-width, -intensity and score might also form the basis for an image-analysis algorithm which mimics the magnification rule. Different approaches have been investigated for HER2-IHC image-analysis by using color intensities [28, 29] or geometric properties of the staining pattern [30]. Recent advances in digital image analysis have shown to increase of inter-observer agreement and decrease of the number of equivocally scored cases [31, 32].

## Conclusions

IHC scoring by using the ‘magnification rule’ is a semiquantitative procedure that circumvents neurophysiological restrictions of our visual system. It is based on physical interrelations and can be used to overcome subjectivity in HER2 IHC-testing, particularly in gastric cancer. It might also be applicable to other membrane-bound IHC-stainings. As a practical and easy-to-use method it has found wide application and was incorporated into national and international recommendation on HER2-IHC [6, 15, 17].

## Additional files

Additional file 1: **Figure S1.** Example photomicrographs of HER2-IHC. Images depict scoring categories 1+, 2+ and 3+ at magnifications reflecting different microscope objectives (2.5× - 63×. Inserts: Magnified details, 4× additional magnification). Note that the linear DAB-precipitates in categories 1+ and 2+ are not perceivable at low power magnification (2.5×, 5×). (TIFF 47314 kb)

Additional file 2: **Figure S2.** Width of HER2 DAB-precipitates and result of in situ hybridization (ISH). Histograms of *n* = 1200 measurements in 40 cases per scoring category; estimated density (graphs). (TIFF 1103 kb)

Additional file 3: **Figure S3.** Scatter-plot of HER2 DAB-precipitates width and color intensity. For scoring intensities 1+ and 2+ (grey), width and intensity show a linear correlation (*r* = 0.73, dashed lined). Scoring category 3+ shows saturated intensity (*n* = 1200 measurements in 40 cases per scoring category). (JPEG 585 kb)

## Abbreviations

ASCO/CAP: American Society of Clinical Oncology / College of American Pathologists
DAB: Diaminobenzidine
HER2: The HER2 protein, i.e. human epidermal growth factor receptor 2
HER2/neu: The HER2/neu gene which encodes the HER2 protein
HERA: Acronym of the clinical phase 3 trial that tested adjuvant Trastuzumab in breast cancer, cf. Lancet. 2017; 389:1195-1205
IHC: Immunohistochemistry
ISH: In-situ hybridization
MR: Magnification rule
NA: Numerical aperture
ROI: Region of interest
SD: Standard deviation
ToGA: Acronym of the clinical phase 3 trial that tested adjuvant Trastuzumab in gastric cancer, cf. Lancet. 2010; 376:687-97
0, 1+, 2+, 3 +: Categories of the scoring system for HER2-IHC interpretation
4B5: Clone of the primary anti-HER2 antibodies used for HER2-IHC
