# Supplementary figures and images for: Physical basis of the ‘magnification rule’ for standardized Immunohistochemical scoring of HER2 in breast and gastric cancer
Source: Diagn Pathol. 2018 Mar 12;13:19. doi: 10.1186/s13000-018-0696-x (PMC5848460; doi:10.1186/s13000-018-0696-x)

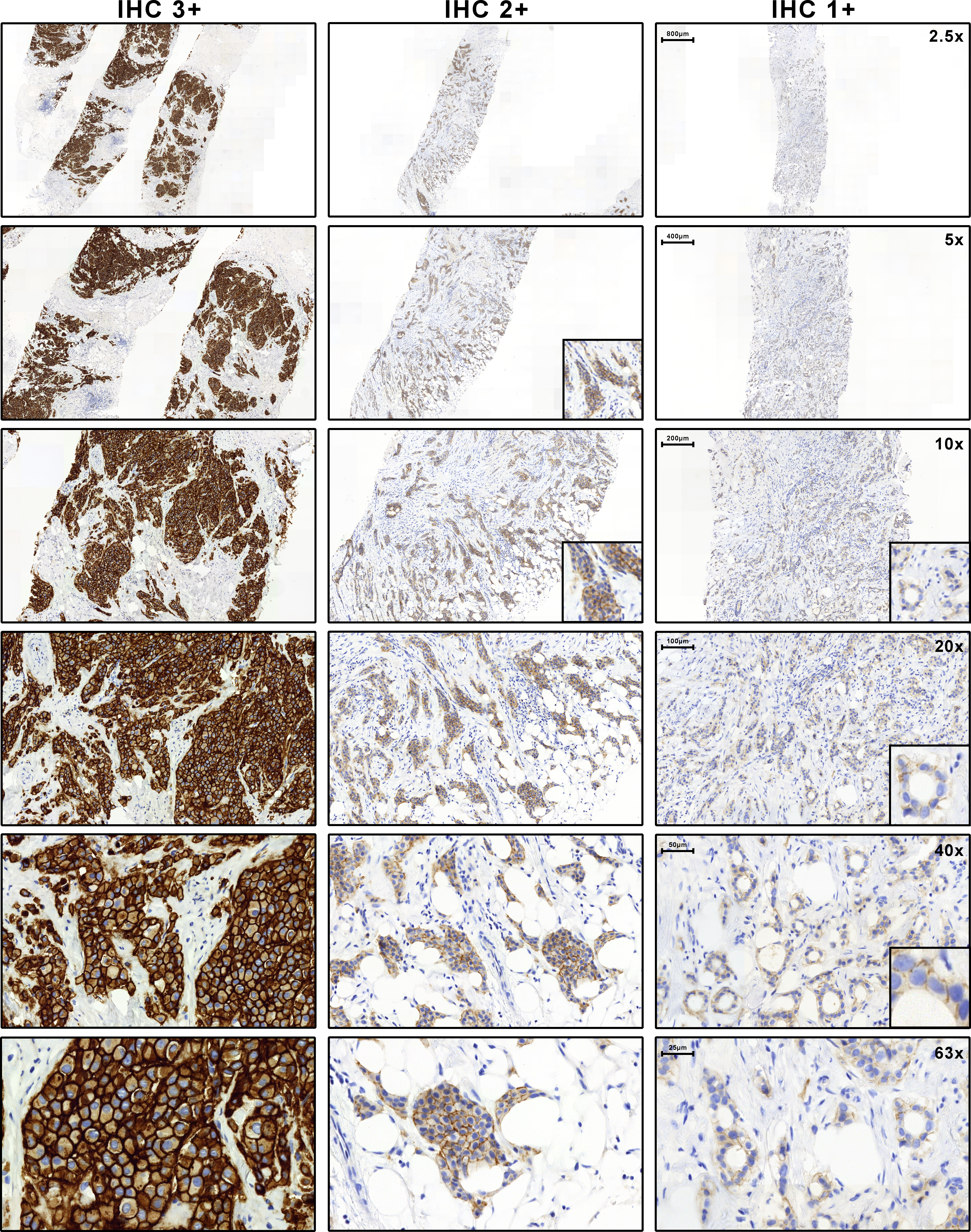

Supplement: Supplementary file 1 — Figure S1. Example photomicrographs of HER2-IHC. Images depict scoring categories 1+, 2+ and 3+ at magnifications reflecting different microscope objectives (2.5× - 63×. Inserts: Magnified details, 4× additional magnification). Note that the linear DAB-precipitates in categories 1+ and 2+ are not perceivable at low power magnification (2.5×, 5×). (TIFF 47314 kb) [file 13000_2018_696_MOESM1_ESM.tif]

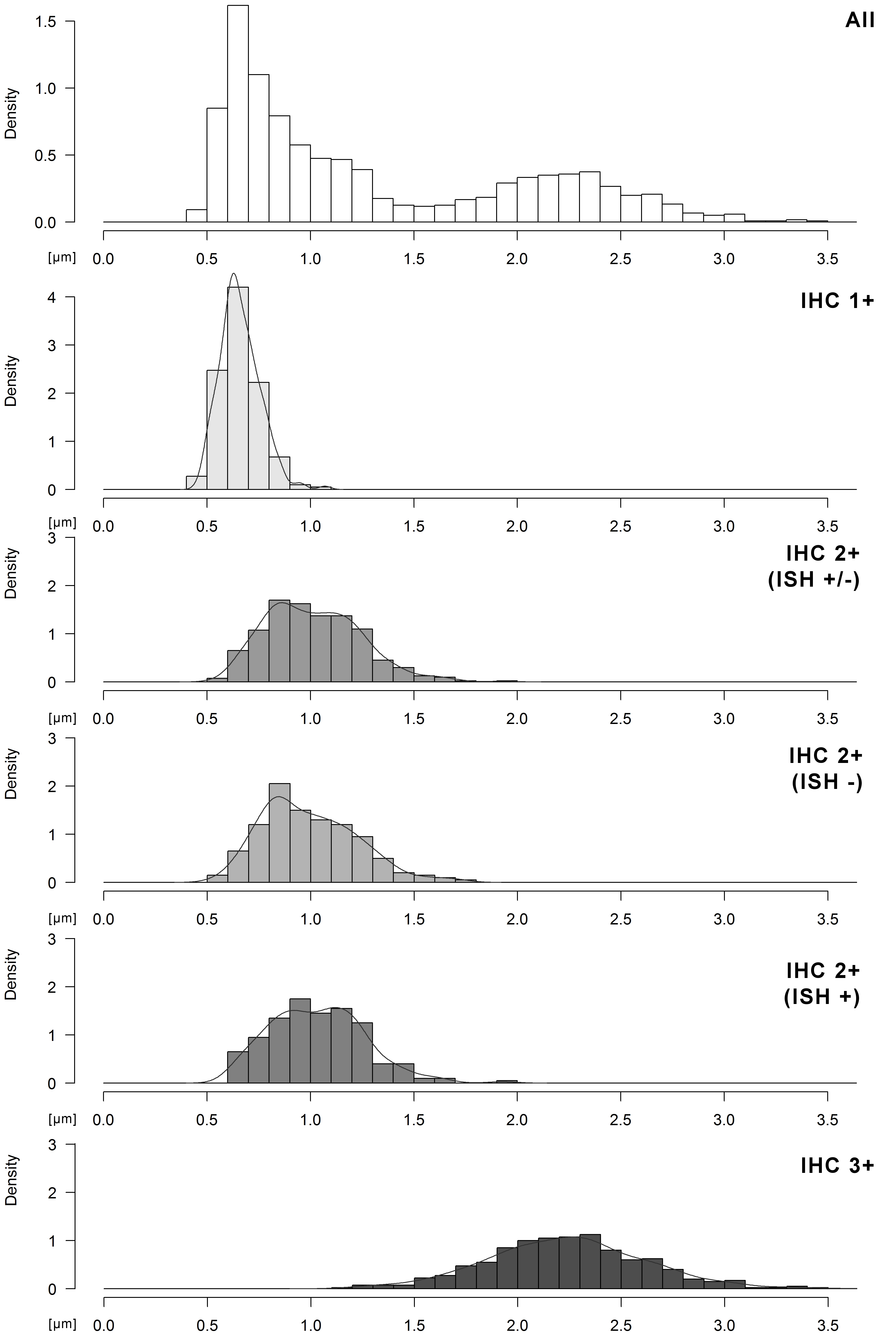

Supplement: Supplementary file 2 — Figure S2. Width of HER2 DAB-precipitates and result of in situ hybridization (ISH). Histograms of n = 1200 measurements in 40 cases per scoring category; estimated density (graphs). (TIFF 1103 kb) [file 13000_2018_696_MOESM2_ESM.tif]

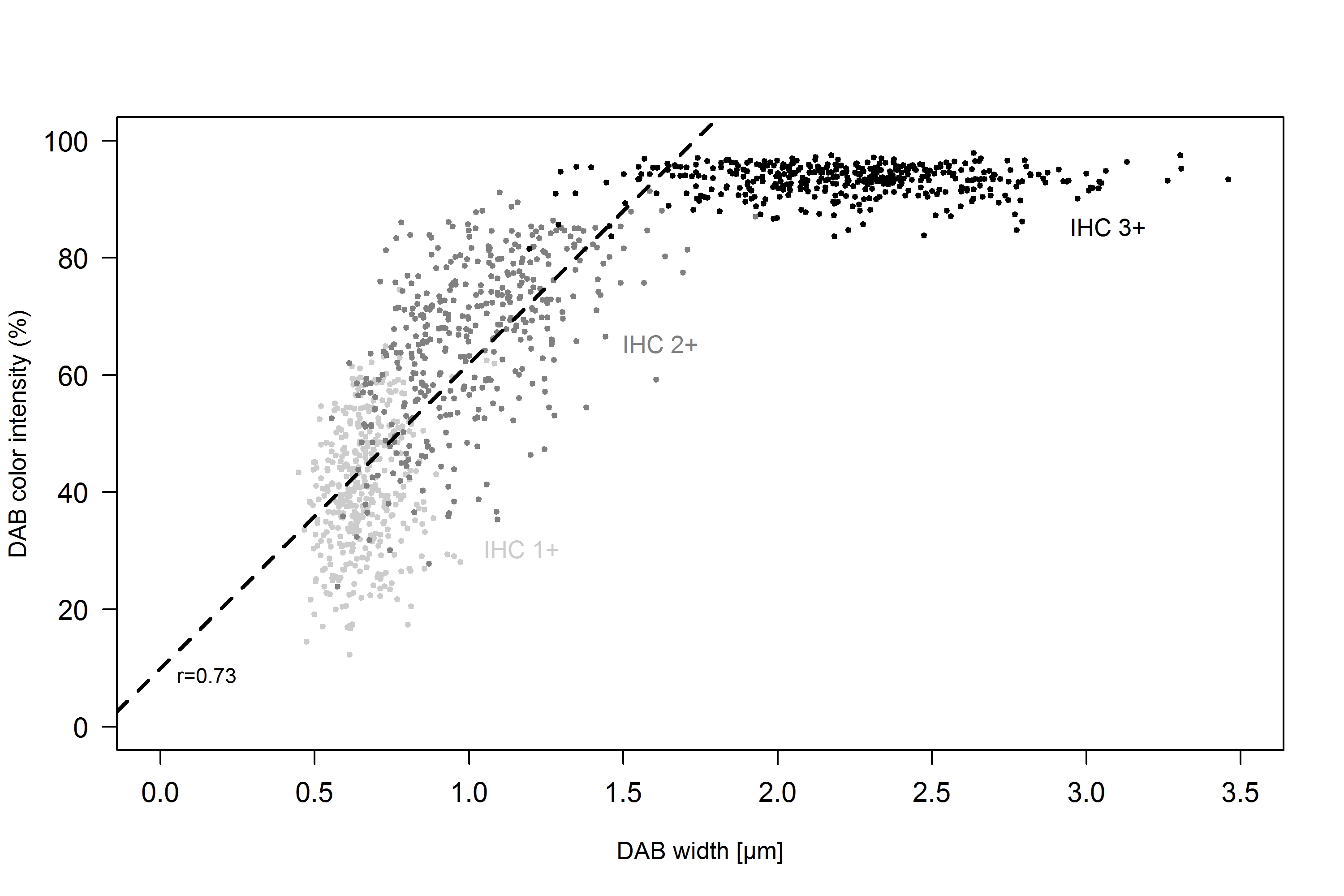

Supplement: Supplementary file 3 — Figure S3. Scatter-plot of HER2 DAB-precipitates width and color intensity. For scoring intensities 1+ and 2+ (grey), width and intensity show a linear correlation (r = 0.73, dashed lined). Scoring category 3+ shows saturated intensity (n = 1200 measurements in 40 cases per scoring category). (JPEG 585 kb) [file 13000_2018_696_MOESM3_ESM.jpg]
